# In-depth insights into cervicovaginal microbial communities and hrHPV infections using high-resolution microbiome profiling

**DOI:** 10.1038/s41522-022-00336-6

**Published:** 2022-09-28

**Authors:** Mariano A. Molina, Karolina M. Andralojc, Martijn A. Huynen, William P. J. Leenders, Willem J. G. Melchers

**Affiliations:** 1 Department of Medical Microbiology, Radboud University Medical Center, 6500 HB Nijmegen, The Netherlands; 2 Department of Medical Microbiology, Radboud Institute for Molecular Life Sciences, Nijmegen, The Netherlands; 3 Department of Biochemistry, Radboud Institute for Molecular Life Sciences, 6525 GA Nijmegen, The Netherlands; 4 Center for Molecular and Biomolecular Informatics, Radboud Institute for Molecular Life Sciences, 6525 GA Nijmegen, The Netherlands; 5 Predica Diagnostics, Toernooiveld 1, 6525 ED Nijmegen, The Netherlands

**Keywords:** Microbiome, Next-generation sequencing, Clinical microbiology

## Abstract

The cervicovaginal microbiome (CVM) correlates with women’s cervical health, and variations in its composition are associated with high-risk human papillomavirus (hrHPV) infection outcomes. Cervicovaginal microbes have been grouped into five community state types (CSTs) based on microbial community composition and abundance. However, studying the impact of CSTs in health and disease is challenging because the current sequencing technologies have limited confident discrimination between closely related and yet functionally different bacterial species. Circular probe-based RNA sequencing (ciRNAseq) achieves high-resolution microbiome profiling and therefore provides in-depth and unambiguous knowledge about the composition of the CVM. Based on ciRNAseq profiling of a large cohort of cervical smears (*n* = 541), we here define subgroups of CSTs I, III, and IV based on intra-CST differences with respect to abundances of *Lactobacillus acidophilus* (CSTs I-A vs. I-B and CSTs III-A vs. III-B)*, Lactobacillus iners* (CSTs I-A vs. I-B and CSTs III-A vs. III-B), and *Megasphaera genomosp type 1* (CSTs IV-A vs. IV-B). Our results further support the existence of subgroups of CST IV-C that are dominant for non-*Lactobacillus* species and have intermediate microbial diversity. We also show that CST V is associated with uninfected conditions, and CST IV-A associates with hrHPV-induced cervical disease. In conclusion, we characterized new subdivisions of cervicovaginal CSTs, which may further advance our understanding of women’s cervical health and hrHPV-related progression to disease.

## Introduction

Human microbiomes play a significant role in health and disease^1,2^. Microbes can colonize several body sites, adapt to the host environment, and develop interdependent associations and communities^3–5^. In particular, the stability of the cervicovaginal microbiome (CVM) is crucial for women’s cervical health. The healthy CVM is dominated by bacteria from the genus *Lactobacillus*, which ensure an acidic environment that protects women against pathogens and opportunistic infections^6–9^. The outgrowth of pathogenic bacteria leads to an imbalance in microbial communities (dysbiosis) that is often associated with disease. An example is bacterial vaginosis (BV), which is characterized by the outgrowth of *Gardnerella vaginalis* and depletion of *Lactobacillus*^10,11^. Cervicovaginal dysbiosis is also associated with miscarriage, preterm birth, and viral infections^12–14^. Microbial communities of the cervicovaginal environment have been grouped into five main community state types (CSTs)^15,16^. However, the current classification of CSTs only partly elucidates the relationship between the microbiota and women’s cervical conditions as these microbiome profiles are highly dependent on bacterial species that cannot be precisely profiled with commonly used sequencing technologies^17^. Hence, it is essential to use sequencing techniques with species-level resolution and high sensitivity to fully understand the impact of the CVM on clinical outcomes and disease.

The earliest classification of microbial communities in the CVM was based on microbial dominance and composition, and the list of defined CSTs has increased over time^15,18^. Originally, Ravel et al. described CSTs I, II, III, and V, which correspond to microbiomes with dominance of *Lactobacillus* species *L. crispatus* in CST-I, *L. gasseri* in CST-II, *L. iners* in CST-III, and *L. jensenii* in CST-V, whereas CST-IV is characterized by a diverse microbial composition^15^. Thereafter, several studies have suggested up to nine CSTs based on dominance of *G. vaginalis* subtypes, and co-occurrence of certain bacterial species^16,19^. This non-uniformity in CST classification impedes the estimation of the correlation of microbial communities with cervical disease in cross-sectional studies^20^. It is therefore essential to establish a consensus in CST classification. For this purpose, France et al. recently developed the VAginaL community state typE Nearest CentroId classifier (VALENCIA) tool to reproducibly assign CSTs in the CVM of reproductive-age women^21^. By applying VALENCIA, the authors observed that women’s CVM clustered in the classical five CSTs^15^, with novel subdivisions being assigned to the well-known CSTs I, III, and IV^21^. Moreover, the study also described that CST IV-C could be further categorized into subgroups based on bacterial dominance^21^. Nonetheless, it is required to determine whether bacterial species support these microbial subgroups by performing high-resolution microbiome profiling. The VALENCIA tool requires amplicon-based 16S rRNA gene sequencing data as input for CST classification^21^. 16S rRNA gene sequencing produces genus-resolution microbiome profiling for many taxa but provides limited species information due to the complexity of the variable regions (VRs) in the 16S rRNA gene^19,22,23^. Alternatively, shotgun metagenomics can perform high-throughput sequencing and allows the determination of microbial communities, species, and strains in the CVM^14^. However, shotgun metagenomics is relatively expensive and it requires specialized resources for computation and data analyses^24^. Thus, to improve CVM profiling, our group recently developed and validated a circular probe-based RNA sequencing (ciRNAseq) technique that achieve high-resolution sequencing and quantification of microbial species by targeting multiple 16S rRNA VRs^25^. Due to barcode technology, CiRNAseq can handle hundreds of samples in one sequencing run, which is cost-effective, and requires fewer specialized skills for data analyses than shotgun metagenomics, making it an accessible technique^25,26^.

CiRNAseq employs single-molecule molecular inversion probes (smMIPs) to target conserved DNA and RNA sequences in the 16S and 18S rRNA genes of microbial species within the CVM. The technique exhibits high specificity and sensitivity in identifying microbial species in mock community samples and women’s cervical smears^25^. Likewise, ciRNAseq provides improved taxonomic resolution compared to 16S rRNA gene sequencing, which is critical for the classification of CSTs and in the study of the association of the CVM with health and disease^23,25^. Application of ciRNAseq on a cohort of cervical smears that were either negative for high-risk human papillomavirus (hrHPV) without cervical lesions or hrHPV-positive with cervical intraepithelial neoplasia 2+ (CIN2+) confirmed the existence of the five classical CSTs and found specific microbiome profiles and bacterial species associated to cervical disease, demonstrating the potential of this sequencing tool for CSTs designation and in elucidating the role of the CVM in hrHPV infections^25^.

Persistent infection with hrHPV can lead to cervical cancer via a progressive series of premalignant stages (CIN). HrHPV-induced cervical aberrations and oncogenesis are associated with changes in the CVM, but the causal interplay between microbial species, CSTs, and hrHPV remains poorly understood^17,27,28^. Characteristically, hrHPV persistence and progression of hrHPV-induced CIN correlate with *Lactobacillus* depletion, an increase in vaginal pH, and colonization by anaerobic bacteria^29^. CST IV, characterized by high microbial diversity, is correlated with hrHPV-induced high-grade cervical lesions, while the low diversity CST I is observed in hrHPV-negative women^27^. Species-level microbiome profiling is crucial to understanding hrHPV-induced CIN. For instance, compared to hrHPV-negative cervices, bacterial species such as *G. vaginalis* and *L. iners* are present at higher and lower relative abundances, respectively, in hrHPV-induced high-grade CIN^16,19,25,30^. Additional species from the genera *Lactobacillus* and *Megasphaera* (e.g., *L. acidophilus*, *M. genomosp type 1,* and *M. micronuciformis*) have been disregarded in most CVM studies, possibly due to the inability to reliably discriminate them by 16S rRNA gene sequencing. Our group and others have previously identified *L. acidophilus* in the CVM of hrHPV-negative women and *M. genomosp type 1* in the CVM of women with hrHPV-induced high-grade CIN^25,31,32^, emphasizing the added value of high-resolution microbiome profiling to investigate the association of the CVM with hrHPV carcinogenesis.

In this study, we employed ciRNAseq for high-resolution CVM profiling to characterize CSTs in a large cohort of samples (*n* = 541) of hrHPV-negative women (*n* = 44) and hrHPV-positive women with (*n* = 200) and without cervical lesions (*n* = 297). Using unsupervised cluster analysis, we illustrate the presence of classical and novel subdivisions of CSTs in the CVM. Evaluation of microbial dominance, abundance, and diversity in these CSTs seemingly implies microbial dynamics reflected by the abundance of *Lactobacillus, Gardnerella*, and *Megasphaera* species that correlate with women’s cervicovaginal conditions. Additionally, we show how this CST classification is relevant in hrHPV-induced cervical disease. Overall, our results show the existence of novel CSTs subdivisions and reveal their association with cervical health and disease.

## Results

### Community state types within the cervicovaginal microbiome

**Fig. 1:**
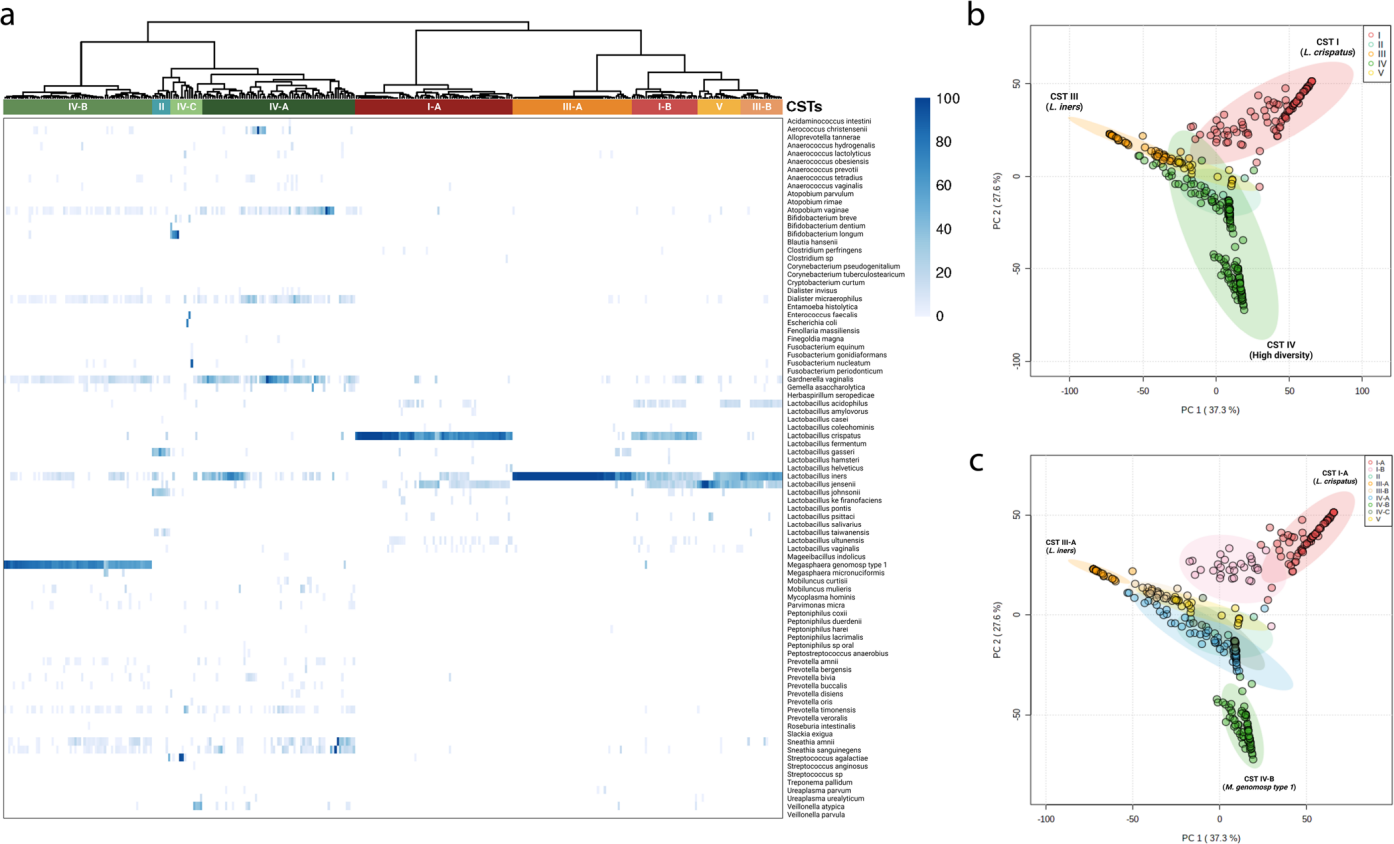
Classical and newest subgroups of community state types within the cervicovaginal microbiome. Unsupervised clustering analysis shows that the CVM from our “Health” cohort (*n* = 341) groups in clusters resembling the classical five CSTs and subclusters consistent with the subclassification for CSTs I, III, and IV. Samples clustered in CSTs according to the dominant bacterium and their microbial composition (I-B, III-B). Clustering distance: Manhattan. Normalized values represent relative abundances (**a**). Separation of our cohort by PC1 and PC2 shows the clusters representing the five traditional CSTs (**b**), and at the same time, such clusters fit within the CST subdivisions (**c**). The microbial species *L. iners* (−0.81) and *L. crispatus* (0.57) have a negative and positive correlation, respectively, with PC1. In contrast, both *L. iners* (0.35) and *L. crispatus* (0.63) show a positive correlation with PC2, while *M. genomosp type 1* (−0.64) and *G. vaginalis* (−0.15) exhibit a negative correlation with PC2 (Supplementary File 1). These species associate with the CSTs III-A, I-A, and IV, respectively, and dominate the PCA distribution (**b**, **c**). Source data are provided as a Source data file. CSTs: community state types.

Here we used ciRNAseq to first profile the CVM from 341 women without cervical abnormalities. Based on the criteria for the “classic classification” of CSTs, 98 women had a CST I (28.7%), eight women a CST II (2.4%), 70 women a CST III (20.5%), 19 women had a CST V (5.6%), and 146 women (42.8%) had a CST IV microbiome. We performed an unsupervised clustering analysis on the samples to examine correlations between ciRNAseq-based high-resolution sequencing results, the existing classification system, and whether potentially clinically meaningful new CST types could be identified (Fig. 1a). Overall, the clustering based on the complete composition of the microbial communities separated into CSTs with high diversity (II and IV) (Fig. 1a, left clusters) and *Lactobacillus*-dominated CSTs (I, III, and V) (Fig. 1a, right clusters)^15,21^.

In addition to characterizing clusters as classical CSTs (Fig. 1b), the clustering identified subgroups within CSTs I and III that were associated with the bacterial species *L. acidophilus* and *L. iners*. Accordingly, CST I was subdivided into I-A (69/98, 70.4%) and I-B (29/98, 29.6%), both dominant for *L. crispatus*, but with I-B exhibiting higher abundance for *L. acidophilus* and *L. iners* (Fig. 1a and Supplementary Table 1). We observed that CST I-B grouped alongside III-A and V, possibly due to their similar *Lactobacillus* species abundance (Fig. 1a). Likewise, CST III was subdivided into III-A (52/70, 74.2%) and III-B (18/70, 25.8%), both dominant for *L. iners*, but with only III-B containing *L. acidophilus*.

**Fig. 2:**
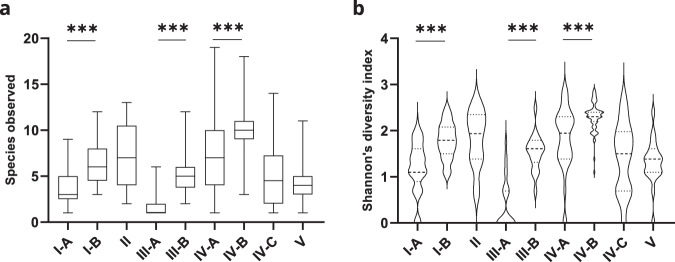
Microbial richness and diversity associate with novel microbiome profiles. **a** Analysis of species richness shows a significantly higher number of species in samples classified as CSTs I-B, III-B, and IV-B compared to I-A, III-A, and IV-A, respectively. **b** Analysis of diversity demonstrates a significantly higher diversity in samples classified as CSTs I-B, III-B, and IV-B compared to I-A, III-A, and IV-A, respectively, as evaluated by Shannon’s index. Error bars represent means ± s.d. and differences in richness and diversity were analyzed by using a Mann–Whitney U test followed by Bonferroni correction. ***p < 0.0001. Source data are provided as a Source data file.

In CST IV, three subgroups were formed, with CST IV-C having a lower diversity than CSTs IV-A and IV-B (Fig. 2a). Non-*Lactobacillus* species dominated CST IV-C while CSTs IV-A and IV-B were dominated by *G. vaginalis*, *L. iners*, *M. genomosp type 1*, and other species listed in Table 1 and Supplementary Table 1. We found 14 women with a CST IV-C (14/146, 9.6%). The subdivision in subclusters CST IV-A and IV-B was caused by the abundance of *M. genomosp type 1*. 67 women had a CST IV-A (67/146, 45.9%), and 65 women had a CST IV-B (65/146, 44.5%), with the latter showing a higher abundance for *M. genomosp type 1* (Fig. 1a). We also analyzed the microbiome composition of women exhibiting CST IV-C. We identified CSTs subdivisions based on bacterial dominance, three of which have been previously described in two studies (IV-C1, IV-C2, and IV-C3)^19,21^ and four of which we classified as novel subgroups: IV-C5, IV-C6, IV-C7, and IV-C8, which exhibited dominance for *E. coli*, *F. nucleatum*, *P. timonensis*, and *V. atypica*, respectively (Table 1).

**Table 1:** Bacterial dominance within CSTs IV-A, IV-B, and IV-C.

Bacterial species	CST IV-A	CST IV-B	CST IV-C	
	*n* (%)	*n* (%)	*n* (%)	Subdivisions
*Aerococcus christensenii*	3 (5)	0 (0)	0 (0)	NA
*Atopobium vaginae*	7 (10)	0 (0)	0 (0)	NA
*A. vaginae/S. sanguinegens*	1 (2)	0 (0)	0 (0)	NA
*Bifidobacterium longum*	0 (0)	0 (0)	3 (21)	IV-C3 (21)
*B. longum/B. dentium*	0 (0)	0 (0)	1 (7)	IV-C3 (21)
*Dialister micraerophilus*	4 (6)	0 (0)	0 (0)	NA
*Enterococcus faecalis*	0 (0)	0 (0)	1 (7)	IV-C2 (21)
*Escherichia coli*	0 (0)	0 (0)	1 (7)	IV-C5
*Fusobacterium nucleatum*	0 (0)	0 (0)	1 (7)	IV-C6
*Gardnerella vaginalis*	28 (42)	0 (0)	0 (0)	NA
*Gemella asaccharolytica*	1 (2)	0 (0)	0 (0)	NA
*Lactobacillus iners*	13 (19)	0 (0)	0 (0)	NA
*Megasphaera genomosp type 1*	1 (2)	64 (98)	0 (0)	NA
*Megasphaera micronuciformis*	0 (0)	1 (2)	0 (0)	NA
*Prevotella timonensis*	0 (0)	0 (0)	1 (7)	IV-C7
*Sneathia amnii*	5 (7)	0 (0)	0 (0)	NA
*Sneathia sanguinegens*	4 (6)	0 (0)	0 (0)	NA
*Streptococcus agalactiae*	0 (0)	0 (0)	2 (15)	IV-C1(21)
*Veillonella atypica*	0 (0)	0 (0)	4 (29)	IV-C8
Total	67	65	14	7

The complete species distribution of this cohort can be found in Supplementary Table 2. The variation of the CVM of the cohort of women with no cervical abnormalities is visualized by a PCA plot (Fig. 1b), where segregation of microbiomes into the five traditional CSTs can be observed. Principal component analysis also separated CST IV-A and CST IV-B (Fig. 1c, blue and green, respectively). CSTs I-A and III-A were separated by PC1, while both CSTs were separated from CST IV-B by PC2. The species of CSTs I-A (*L. crispatus*), III-A (*L. iners*), and IV-B (*M. genomosp type 1*) dominated the loadings of the PCA axes (Fig. 1c and Supplementary File 1).

We then calculated species richness (Fig. 2a) and alpha diversity (Fig. 2b) indices for all CSTs. Both indices were significantly higher in CSTs I-B, III-B, and IV-B compared to I-A, III-A, and IV-A, respectively, further supporting the CST subdivisions (Fig. 2). In conclusion, traditional cervicovaginal CSTs can be further classified into subgroups based on high-resolution microbial composition analysis.

### Microbial abundances in novel CST subdivisions

**Fig. 3:**
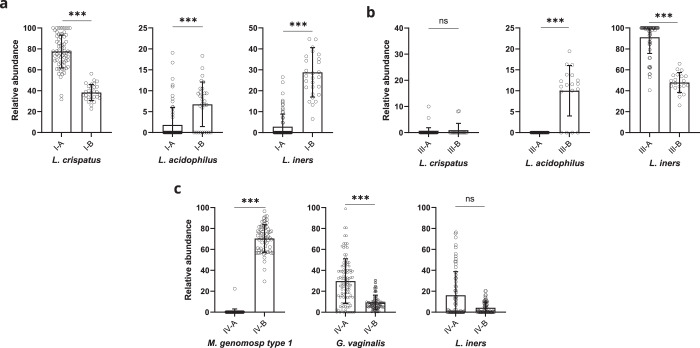
Association of microbial relative abundances with CSTs. **a** Analysis of species relative abundances in CST I-A and I-B reveals a higher significant abundance for *L. crispatus* in I-A compared to I-B and for *L. iners* and *L. acidophilus* in I-B compared to I-A. **b** Analysis of CSTs III-A and III-B shows a significantly higher abundance for *L. iners* in III-A compared to III-B and for *L. acidophilus* in III-B compared to III-A. **c** Analysis of CSTs IV-A and IV-B demonstrates a significantly higher abundance for *M. genomosp type 1* in IV-B than IV-A and for *G. vaginalis* in IV-A than IV-B. Error bars represent means ± s.d. and differences in relative abundances were analyzed by using a Mann–Whitney U test followed by Bonferroni correction. *p < 0.005; **p < 0.001; ***p < 0.0001; ns, not significant. Source data are provided as a Source data file.

Relative abundances of the most abundant species were determined in the new CST subgroups. The abundance of *L. crispatus* was higher in CST I-A than in I-B (*p* < 0.0001) but similar in III-A and III-B (*p* = 0.4924) and the other CSTs (Fig. 3a, b, Supplementary Fig. 1). *L. iners* was more abundant in CSTs I-B, III-A, and V when compared to I-A (*p* < 0.0001), III-B (*p* < 0.0001), and II (*p* = 0.0263), respectively (Fig. 3a, b, Supplementary Fig. 1). *L. acidophilus* was significantly more abundant in CSTs I-B, III-B, and V than in I-A (*p* < 0.0001), III-A (*p* < 0.0001), and IV-A (*p* < 0.0001), respectively (Fig. 3a, b, Supplementary Fig. 1). Additional analysis of other *Lactobacillus* species showed that *L. jensenii* was more abundant in CST I-B and III-B than I-A (*p* = 0.0103) and III-A (*p* < 0.0001), respectively (Supplementary Fig. 1a). As expected, *L. jensenii* and *L. gasseri* were more abundant in CSTs V and II, where these species dominate the microbiome (Supplementary Fig. 1).

**Fig. 4:**
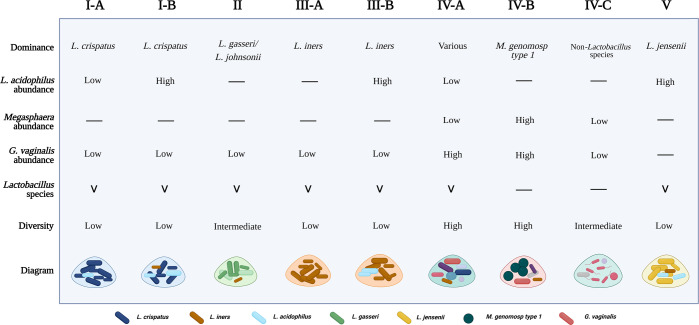
Characteristics of novel cervicovaginal microbial community state types. CST I-A exhibits *L. crispatus* dominance, low abundance of *L. acidophilus*, and low diversity. CST I-B also possesses *L. crispatus* dominance but a higher abundance of *L. acidophilus* and *L. iners*. CST II retains *L. gasseri/L. johnsonii* dominance and has low diversity. CST III-A owns dominance for *L. iners* and shallow diversity. CST III-B is also characterized by *L. iners* dominance but includes *L. acidophilus*. CST IV-A displays dominance for various species, high abundance of *G. vaginalis*, and low abundance of *M. genomosp type 1*. CST IV-B is characterized by dominance for *M. genomosp type 1*, colonization by *G. vaginalis*, and *Lactobacillus* depletion (excluding *L. iners*). CST IV-A and IV-B have a high diversity. CST IV-C is dominated by non-*Lactobacillus* species and exhibits *Lactobacillus* depletion with intermediate diversity. CST V has *L. jensenii* dominance, high abundance for *L. acidophilus* and *L. iners*, and overall low diversity. Presence of microbes is indicated by (V). Absence of microbes is indicated by (−). Non-*Lactobacillus* species and “Various” species that exhibit dominance in CSTs IV-A and IV-C, are listed in Supplementary Table 1. Created with BioRender.com.

Next, we analyzed the relative abundances for *M. genomosp type 1*, *G. vaginalis*, and *L. iners* in CST IV. The abundance for *G. vaginalis* was significantly lower in IV-B than in IV-A (*p* < 0.0001), but for *L. iners*, this difference was not significant (*p* = 0.1149) (Fig. 3c). Other bacteria such as *A. vaginae* (*p* = 0.0015) and *D. micraerophilus* (*p* = 0.0003) also exhibited a significantly higher abundance in CST IV-A compared to IV-B (Supplementary Fig. 2), while the relative abundance of species *S. amnii* (*p* = 0.3681), *S. sanguinegens* (*p* = 0.0605), *P. timonensis* (*p* = 0.5166), and *M. mulieris* (*p* = 0.5551) did not significantly vary between CSTs IV-A and IV-B (Supplementary Fig. 2, Source data file). The presented significant differences in microbial abundances between new CSTs further illustrate the characteristics of the novel subclassification. It also indicates the bacterial species relevant for subdivision. The key features of newly defined CSTs are summarized in Fig. 4, and a descriptive comparison with previous CST classifications is shown in Supplementary Fig. 3.

### Microbial communities in hrHPV-associated cervical disease

**Fig. 5:**
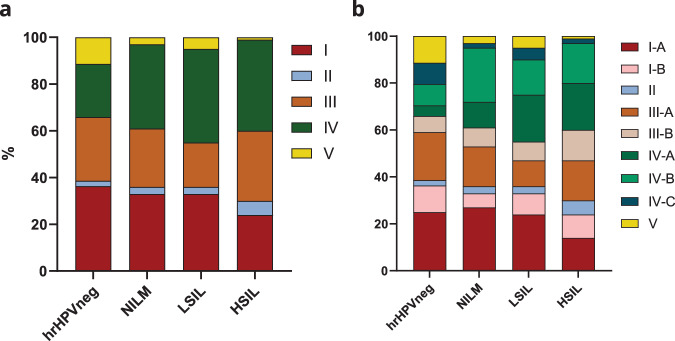
Distribution of microbial communities in hrHPV-associated cervical disease. **a** The CVM in hrHPV-negative women and hrHPV-positive women with no cervical lesions (NILM), low-grade cervical lesions (LSIL), and high-grade cervical lesions (HSIL) illustrate the microbial dynamics occurring upon hrHPV infection and disease progression according to the classical five CSTs. These dynamics reflect the well-described decrease in CST I and increase in CST IV. There is also a significant decrease in CST V. Analysis of the same cohort considering the subdivision of CSTs shows the reduction of CST I observed in (**a**) from hrHPV-negative to HSIL is associated with CST I-A (**b**) while CST III-A seems stable in all stages. Alternatively, there is a slight increase in the proportion of women with CST II and III-B in HSIL compared to NILM (**b**). CST IV-A likely occurs upon hrHPV infection (**b**), and its incidence increases upon disease progression (LSIL and HSIL). hrHPVneg: hrHPV-negative, *n* = 44; NILM*, n* = 100; LSIL, *n* = 100; HSIL, *n* = 100. Source data are provided as a Source data file.

To investigate whether CSTs correlated with disease, we studied the prevalence of CST subgroups in a “disease stages” cohort of hrHPV-negative women (*n* = 44) and hrHPV-positive women diagnosed with NILM (*n* = 100), LSIL (*n* = 100), and HSIL (*n* = 100) (Fig. 5) and evaluated their association through a Fisher’s exact test.

We observed the classical CST groups in all stages of cervical dysplasia, but with a distinctive occurrence in women with low- and high-grade cervical lesions. When compared to hrHPV-negative women, we found a 1.6x increase in occurrence of CST IV for the NILM group (*p* = 0.126), 1.8x for LSIL (*p* = 0.057), and 1.7x for HSIL (*p* = 0.084). Likewise, we also observed the distinctive decrease of *L. crispatus* dominance (CST I) from NILM throughout LSIL and HSIL (Fig. 5a)^28^. Interestingly, we observed a decrease in CST V in hrHPV-positive women, particularly in HSIL, with an 11x reduced prevalence compared to the hrHPV-negative group (*p* = 0.0104), indicating that CST V could be associated with uninfected conditions. In contrast, we found a 2.7x higher prevalence of CST II in HSIL than in the hrHPV-negative group (*p* = 0.675) (Fig. 5a).

There were major differences in the prevalence of CSTs in the various disease stages when considering the novel microbial subgroups. The frequency of CST I women containing *L. acidophilus* (CST I-B) was highest in the hrHPV-negative group compared to hrHPV-positive women with NILM (*p* = 0.311). In contrast, the occurrence of CST I-A women was highest in NILM when compared to HSIL (*p* = 0.0509). This analysis shows dynamics within CST I in different stages of hrHPV-related disease (Fig. 5b). We also observed a consistent prevalence of CST III presenting women throughout hrHPV infection and cervical lesions (Fig. 5a), with CST III-B (Fig. 5b) resembling the frequency of CST I-B women (both characterized by the presence of *L. acidophilus*). The HSIL group showed a 1.9x higher prevalence of CST III-B compared to hrHPV-negative women (*p* = 0.391). HrHPV-negative women had 4.4x reduced prevalence of CST IV-A, whereas its occurrence was particularly high in women with LSIL (*p* = 0.021) and HSIL (*p* = 0.059) (Fig. 5b). Similarly, CST IV-B was 2.6x more prevalent in hrHPV-positive women with NILM than in hrHPV-negative women (*p* = 0.063). The increase in CST IV-A prevalence within LSIL and HSIL and the high frequency of CST IV-B-presenting women hrHPV-positive highlights the association of CST IV with hrHPV-induced cervical lesions previously described^25^. Likewise, CST V associates with healthy uninfected conditions, while CST I-A associates with the absence of cervical abnormalities. In conclusion, the subdivision of microbial communities in the CVM shows interesting microbial dynamics that may correlate with hrHPV-associated infection and cervical lesions.

## Discussion

Using high-resolution microbiome profiling, our study confirmed the existence of the newly defined classification of CSTs^21^, elucidating the dynamics of the CVM during health and disease. By analyzing CSTs in hrHPV-positive women, we have shown that specifically, CST IV-A associates with hrHPV infection in both low- and high-grade cervical lesions, while CST V is typical for hrHPV-negative women^25^. Our data indicate that classifying cervicovaginal CSTs into five major groups provides a better understanding of microbial communities that correlate with cervical disease than broadening the classification beyond five main groups. This way, we distinguished communities such as I-B, III-B, and IV-A that could be considered transitional profiles between CSTs I, III, and V (I-B and III-B) and between CSTs III and IV (IV-A). Nonetheless, considering our current results, it remains challenging to hypothesize about the direction of microbial shifts. To elucidate such dynamics appropriate longitudinal studies will be essential.

Previously, Brooks et al. reported the existence of nine CSTs, recommending the classification of CSTs VI to IX based on the analyses of five CVM datasets obtained through 16S rRNA gene sequencing and shotgun metagenomics^19^. More recently, France et al. suggested re-classifying cervicovaginal microbiome communities into five major CSTs and their subdivisions^21^. Like France et al., we observed that CST II resembles CST IV, particularly IV-C, which could be associated with their similar diversity and the high vaginal pH found in women exhibiting these two CSTs^21^. Our analyses also indicate that CSTs VI and VIII, described by Brooks et al.^19^, could be combined into CST IV-C. CST IV-C is typically dominated by non-*Lactobacillus* bacteria and possesses low to intermediate diversity compared to CSTs IV-A and IV-B. Hence, CST IV-C could function as an umbrella CST for microbiome profiles dominated by bacterial species from the genera *Fusobacterium*, *Prevotella*, *Streptococcus*, *Escherichia*, *Bifidobacterium*, *Enterococcus*, and others, but which lack the composition and diversity characteristic of CSTs IV-A and IV-B^21,33,34^. Curiously, in a study exploring the association of the CVM with preterm birth, Feehily et al. distinguished CST IV-C with *Bifidobacterium* dominance (IV-C3) and CST IV-A, demonstrating that these novel microbial communities can also be identified with high-throughput sequencing methods such as shotgun metagenomics^14^. Furthermore, we suggest a re-classification of CST IX^19^ to CST IV-A, which has been generally characterized by a co-occurrence of *G. vaginalis* and *L. iners*^35^. Interestingly, women with CST IV-A exhibit colonization by anaerobic bacteria such as *S. amnii*, *S. sanguinegens*, *D. micraerophilus*, and *A. vaginae*, and lower abundance for *M. genomosp type 1*. Once *M. genomosp type 1* colonizes the vagina and becomes dominant, *Lactobacillus* species are fully depleted, and the CVM is characteristic of a CST IV-B. These observations are in line with Albert et al., who described a subdivision of CST IV in IV-C and IV-D based on subtypes of *G. vaginalis*^36^. These two subgroups are characterized by the co-occurrence of *G. vaginalis* and *M. genomosp type 1* and, therefore, fit with CST IV-B^21,36^. In addition, our findings are further supported by the increasing evidence that *Megasphaera* species play a harmful role in women’s cervical health^37–40^. These overall results imply microbial dynamics within CST IV and represent an advantage for understanding the CVM in cross-sectional studies.

The occurrence of *L. acidophilus* in the CVM was particularly observed in CSTs dominant for *Lactobacillus*. Here we report that *L. acidophilus* abundance indicates a distinct microbial state and thus, supports the subdivision of CSTs I and III. These results are consistent with the CST classification by France et al. and may further explain the lower abundance of *L. crispatus* and *L. iners* observed in CSTs I-B and III-B, respectively^21^. In fact, our findings hint that *L. acidophilus* is possibly associated with the abundance of specific bacterial species. This finding agrees with studies that have shown that *L. acidophilus* occurrence associates with a healthy cervix and a protective immune response, which may indicate its ability to enhance beneficial *Lactobacillus* species such as *L. crispatus*^41–44^. Alternatively, *L. acidophilus* occurrence may also lead to a decrease in the abundance of *Lactobacillus* species that generally dominate the CVM, facilitating the colonization by other microbes, an increase in diversity, and the transition to a different CST. *L. acidophilus* has been disregarded in most CVM studies because it is closely related to other species. Previous research has demonstrated that *L. gasseri*, *L. johnsonii*, and *L. acidophilus* belong to the same *Lactobacillus* complex and have a high level of identity in the 16 rRNA gene^45,46^. Such level of identity represents a challenge for 16S rRNA gene sequencing because the technique may not be able to distinguish *L. acidophilus* from *L. gasseri/L. johnsonii* or *L. acidophilus* from *L. crispatus*, as described by several reports^47–49^. Consequently, this might have resulted in identifying other *Lactobacillus* species instead of *L. acidophilus*, which could be explained by their frequent co-occurrence described in the present study. Since ciRNAseq can perform high-resolution microbiome profiling with high specificity and sensitivity, these results further validate the potential of ciRNAseq for exploring the CVM and its microbial interactions at the species taxonomic level^23,25,49,50^. Furthermore, our findings detecting *L. acidophilus*-associated CSTs (I-B and III-B) in low- and high-grade cervical lesions partially agree with Kwasniewski et al., who reported the bacterium in hrHPV-induced lesions^51^. The study found a high incidence of *G. vaginalis* and *L. acidophilus* in HSIL patients, which is in line with our results on CSTs I-B and III-B in this group. However, the Kwasniewski study did not find *L. acidophilus* in healthy conditions, as observed in our latest investigations^25,51^. Thus, additional studies are needed to clarify the role of *L. acidophilus*-containing CSTs in hrHPV-induced carcinogenesis.

**Fig. 6:**
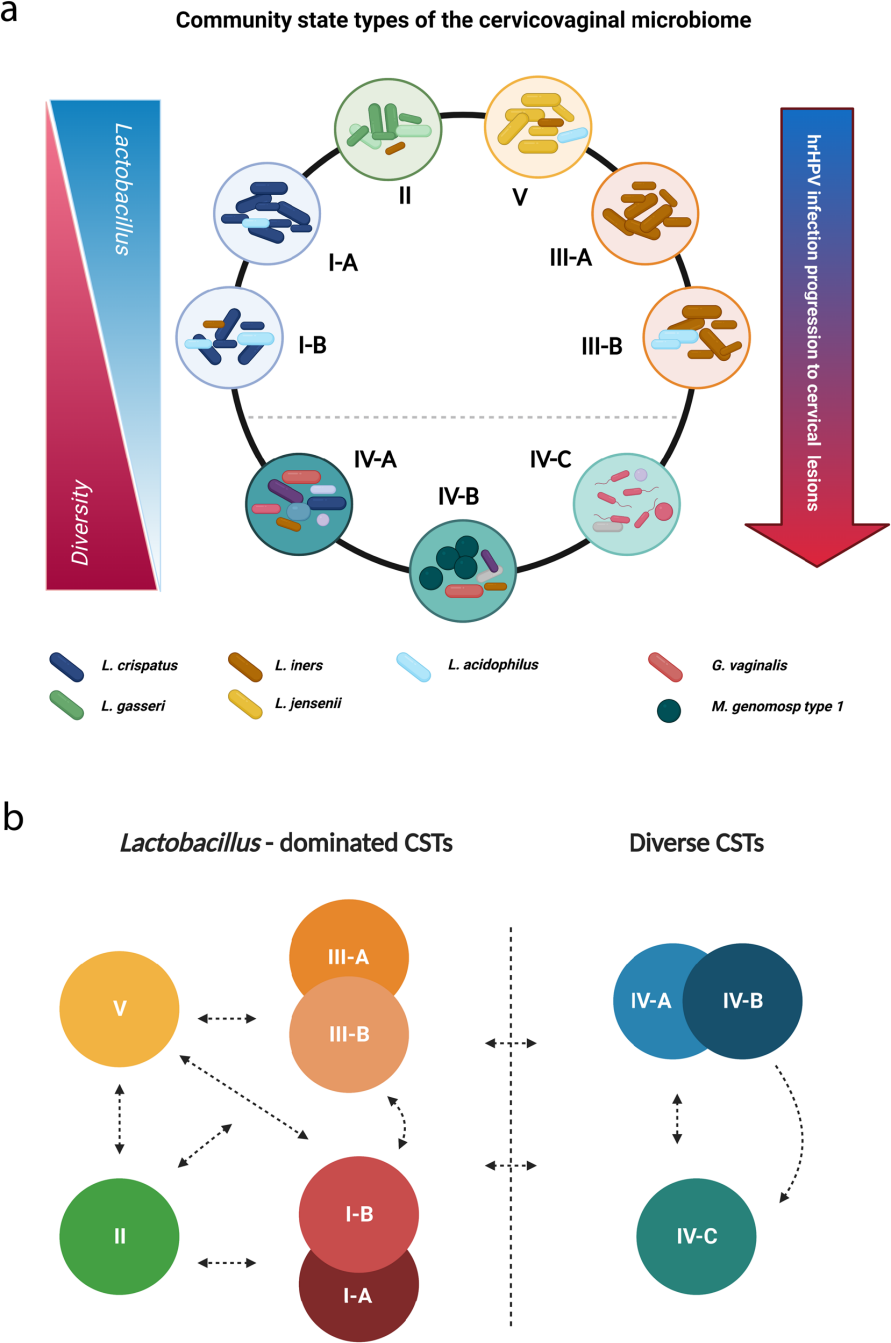
Proposed model for microbial dynamics in health and disease. **a** During “healthy” cervical conditions, the CVM is prominently characterized by *Lactobacillus* dominance and low diversity. Upon hrHPV infection, diversity in means of CST IV increases, and *Lactobacillus*-dominated CSTs decrease, exacerbating hrHPV persistence and disease progression. **b** Classical *Lactobacillus*-dominated CSTs include I, II, III, and V. CSTs I and III also include the subgroups I-A/I-B and III-A/III-B, respectively. The dynamics between CST II, III, and V remain enigmatic, and we hypothesize that the CVM can shift between each state. Alternatively, CST I-B seemingly acts as a transitional state between I-A and III. The characteristic increased diversity in I-B may then lead to a transition to CSTs III-B, IV-A, and V. Similarly, CST III-B may also transition to CSTs I-B, IV-A, and V. The highly diverse CSTs are IV-A, IV-B, and IV-C. CST IV-A can acquire *M. genomosp type 1* dominance and *Lactobacillus* depletion, which leads to IV-B, or lose its diversity and transition to IV-C and *Lactobacillus*-dominated CSTs. Likewise, CST IV-B might also shift to IV-A or lose diversity and *G. vaginalis* colonization, resulting in CST IV-C. CST IV-C can gain diversity and shift to IV-A or transition to *Lactobacillus*-dominated CSTs. Created with BioRender.com.

Our work supports a model where microbial diversity increases upon hrHPV infection and progression of hrHPV-associated lesions and where regression of cervical lesions and hrHPV infections result in a lower number of species in the CVM^17,27,28^ (Fig. 6a). In addition, our data may explain microbial dynamics occurring in CSTs^16,35,52^. The observed abundance of *L. acidophilus* in *Lactobacillus*-dominated CSTs (Figs. 2 and 4), specifically I-B, III-B, and V, might demonstrate a previously unknown shifting mechanism within these CSTs (Fig. 6b). Considering our analyses, we can also hypothesize other dynamics between CSTs I, II, III, and V (Fig. 6b). Upon cervicovaginal dysbiosis, certain bacteria colonize the CVM, resulting in a shift to CST IV (Fig. 6b). Our study also reported bacterial associations within CSTs IV-A and IV-B, and we propose dynamics that may explain these microbial interactions (Fig. 6b).

The strength of the study is the use of high-resolution microbiome profiling for targeting the cervicovaginal microbiota at both DNA and RNA levels. CiRNAseq also provides information on microbial gene expression and metabolic activities, offering more insights into microbial abundances in the complex microbiome. Potential limitations include using a defined panel to characterize the CVM, therefore missing unidentified microbial species. In addition, the use of hrHPV-positive cervical smears in our “health” cohort might have caused a higher observation of CSTs typical during hrHPV infections such as IV-A and IV-B. Nonetheless, this particular cohort was only employed to classify communities, thus not affecting our findings on novel CSTs. Although we also observed these new CSTs in our small cohort of hrHPV-negative women, further studies with a larger cohort of hrHPV-negative cervical smears will be necessary to investigate these communities during healthy cervical conditions. Moreover, our study did not include supplementary information about our participants, such as ethnicity, cervicovaginal pH, and Nugent scoring, which are known factors that correlate with CSTs^15,16,18,19,21,35,36,53^.

In summary, our research promotes an agreement on CSTs designation based on high-resolution CVM profiling, considering microbial dominance, composition, abundance, and diversity. More notably, this classification suggests microbial dynamics occurring in the CVM. Our data emphasize the microbial identification of commonly overlooked bacterial species such as *L. acidophilus* and *M. genomosp type 1*, relevant for cervical health, microbial relationships, and dynamics, and which require high-resolution microbiome profiling for adequate classification. In addition, it is plausible that other known or unknown bacteria and strains could lead to further CSTs classifications. Nevertheless, our study endorses the designation of five main CSTs where transitional or rare CSTs should be assigned in subdivisions. Although we analyzed such subdivisions in the context of hrHPV infections, more studies are required to explain their importance in disease. Taken together, our research shows new insights into microbial communities and their association with hrHPV infections.

## Methods

### Ethics statement

The Central Committee on Research Involving Human Subjects (CCMO), the local Board of Directors of Radboudumc (RvB), and the National Institute for Public Health and Environment (RIVM) reviewed and granted approval before the start of the study (No. 2014-1295). All methods were performed in accordance with the Radboudumc ethical guidelines for using human samples, including the Declaration of Helsinki.

### Study participants and samples

For this study, a total of 541 cervical smears in PreservCyt were collected from women participating in the Dutch population-based cervical cancer screening program and that were received and processed for hrHPV detection. Women participating in the screening were informed that residual material could be used for anonymous research and had the opportunity to opt-out. Only residual material from women who did not opt-out was included. Written and informed consent was obtained from each subject and was required for participation. The histological follow-up outcomes were obtained from the nationwide network and registry of histo- and cytopathology in the Netherlands (PALGA; Houten, the Netherlands). One set of 341 cervical smears that were either hrHPV-negative (*n* = 44) or hrHPV-positive (*n* = 297) with normal cytology (negative for intraepithelial lesion or malignancy, NILM) was used for the analysis of CSTs in healthy conditions. The remaining 200 hrHPV-positive cervical smears were from women diagnosed with low-grade squamous intraepithelial lesions (LSIL, *n* = 100) and high-grade squamous intraepithelial lesions with cervical intraepithelial neoplasia 2 or higher (CIN2+, HSIL, *n* = 100). From the 297 hrHPV-positive cervical smears with normal cytology, we also randomly selected 100 samples to study microbial communities during hrHPV infections and compare their frequency with the LSIL and HSIL groups. Five milliliters of each cervical cell suspension were centrifuged for 5 min at 2500 × *g*, and the pellet dissolved in 1 ml of Trizol reagent (Thermo Scientific). RNA was isolated through standard procedures and dissolved in 20 μl nuclease-free water. We routinely processed a maximum of 2 μg of RNA for DNase treatment and cDNA generation, using SuperscriptII (Thermo)^25^.

### HrHPV identification and genotyping

DNA from all cervical smears was isolated using MagNA Pure (Roche, Bazel, Switzerland). The purified DNA was eluted in 50 μl TE-buffer^54^. Thereafter, broad-spectrum hrHPV amplification was performed using the short-PCR-fragment line probe assay (SPF10-LiPA25; Labo Bio-medical Products B.V., Rijswijk, The Netherlands). This assay amplifies a small fragment of 65 bp from the L1 open reading frame and allows detection of a broad range of HPV genotypes with high sensitivity^55^. The HPV genotypes 16, 18, 31, 33, 35, 39, 45, 51, 52, 56, 58, 59, 66, 68, and 73, considered oncogenic types by the World Health Organization, were characterized as hrHPV in this study.

### CiRNAseq and output analysis

High-resolution microbiome profiling was performed on ~50 ng of cDNA/DNA using the ciRNAseq technology^25,56^. During ciRNAseq, smMIPs bind to multiple VRs in the 16S and 18S rRNA genes of microbial species within the CVM. Following capture hybridization, probe circularization, and purification, circularized probes were subjected to PCR with barcoded Illumina primers. After purifying the correct-size amplicons, quality control, and quantification^54^, a 4 nM library was sequenced on the Illumina Nextseq500 platform (Illumina, San Diego, CA) at the Radboudumc sequencing facility. Reads were mapped against reference regions of interest in our Cervicovaginal Microbiome Panel containing 321 microbial species using the SeqNext module of JSI Sequence Pilot version 4.2.2 build 502 (JSI Medical Systems, Ettenheim, Germany). The settings for read processing were a minimum of 50% matching bases, a maximum of 15% mismatches, and a minimum of 50% consecutive bases without a mismatch between them; for read assigning, the threshold was a minimum of 95% of identical bases with the ROIs. All identical PCR products were reduced to one consensus read (unique read counts, URC) using a unique molecular identifier. We set an arbitrary threshold of at least 1000 URC from all smMIPs combined in an individual sample, below which we considered an output non-interpretable. For microbial annotation, species with two reactive smMIPs were annotated when 100% of the specific set of smMIPs had URC. Species with three or more reactive smMIPs were annotated when more than 50% of their specific set of smMIPs had URC^25^.

### Microbiome classification and assessment

Dominance of microbial species was defined by highest relative abundance. CSTs were classified into I, II, III, IV, and V as described by Ravel et al. and France et al.^15,21^, who defined microbial communities based on microbiome composition. We next performed unsupervised clustering analyses and compared the clusters to known CSTs. Microbiomes that fulfilled the initial classification based on dominance and exhibited distinct microbiome composition in separate clusters were considered new subgroups.

### Statistical analysis

Hierarchical clustering (HC) and Principal component analyses (PCA) were performed using ClustVis^57^ and MetaboAnalyst v5.0^58^. The settings for HC were as follows: clustering distance for columns: Manhattan^59^; clustering method: Ward^60^. GraphPad Prism v9.1.2 (GraphPad Software, Inc., USA) was used to analyze datasets and determine Species richness and Shannon’s diversity index. The statistical significance of differences was calculated using the Mann–Whitney U test. For evaluating microbial richness, diversity, and abundance, we applied the Bonferroni correction for multiple comparisons^61^.

## Supplementary information

Supplementary Information

Supplementary File 1 - PCA loadings

Accession codes

## Data Availability

All data generated or analyzed during this study are included in this published article and its supplementary information files or are available from public repositories. The sequence read data generated in this study are available at NCBI in the Sequencing Read Archive, project PRJNA856437 (452 files)^62^, and at EMBL in the European Nucleotide Archive, project PRJEB45937 (89 files, Sample Accession Numbers are shown in Supplementary File 2)^63^. Source data are provided with this paper.

## Source data

Source Data file
