# Supplementary material for: In-depth insights into cervicovaginal microbial communities and hrHPV infections using high-resolution microbiome profiling
Source: NPJ Biofilms Microbiomes. 2022 Sep 28;8:75. doi: 10.1038/s41522-022-00336-6 (PMC9519886; doi:10.1038/s41522-022-00336-6)
Supplement: Supplementary file 1 — Supplementary Information [file 41522_2022_336_MOESM1_ESM.pdf]

Supplementary Information files for:

In-depth insights into cervicovaginal microbial communities and hrHPV infections using high-resolution microbiome profiling

Mariano A. Molina[1,2,*], Karolina M. Andralojc[1,3,*], Martijn A. Huynen[4], William P. J. Leenders[3,5,b], Willem J. G. Melchers[1,b,†]

[1]Department of Medical Microbiology, Radboud University Medical Center, 6500 HB, Nijmegen, The Netherlands.

[2]Department of Medical Microbiology, Radboud Institute for Molecular Life Sciences, Nijmegen, The Netherlands.

[3]Department of Biochemistry, Radboud Institute for Molecular Life Sciences, 6525 GA, Nijmegen, The Netherlands.

[4]Center for Molecular and Biomolecular Informatics, Radboud Institute for Molecular Life Sciences, 6525 GA, Nijmegen, The Netherlands.

[5]Predica Diagnostics, Toernooiveld 1, 6525 ED, Nijmegen, The Netherlands.

[*]These authors contributed equally.

[b]These authors jointly supervised this work.

[†]Corresponding author: Willem J. G. Melchers, Willem.Melchers@radboudumc.nl

Supplementary Table 1. Classification criteria for community state types.

CSTs	Classification criteria			
	**1. Dominance**	**2. Composition[c]**	**3. Abundance[d]**	**4. Diversity index, 95% CI**
**I-A**	*L. crispatus*			1.197 [1.062, 1.331]
**I-B**	*L. crispatus*		High for *L. acidophilus* and *L. iners*	1.782 [1.635, 1.930]
**II**	*L. gasseri/L. johnsonii*			1.812 [1.289, 2.336]
**III-A**	*L. iners*			0.411 [0.254, 0.567]
**III-B**	*L. iners*	*L. acidophilus*	High for *L. jensenii*	1.534 [1.338, 1.730]
**IV-A**	Various[a]	*G. vaginalis*		1.824 [1.656, 1.993]
**IV-B**	*M. genomosp type 1*	*G. vaginalis*	Low for *Lactobacillus*	2.305 [2.226, 2.384]
**IV-C**	Non-*Lactobacillus* species[b]			1.414 [0.972, 1.856]
**V**	*L. jensenii*		High for *L. acidophilus* and *L. iners*	1.314 [1.075, 1.552]

[a]Bacterial species from the genera *Gardnerella, Lactobacillus, Sneathia, Atopobium, Streptococcus, Aerococcus, Dialister, Veillonella*, and others.
[b]Bacterial species from the genera *Fusobacterium*, *Prevotella*, *Streptococcus*, *Escherichia*, *Bifidobacterium*, *Enterococcus*, and others.
[c]Bacterial species that colonize the CVM and correlate with microbial subgroups.
[d]Characteristic abundances of bacterial species in CSTs subgroups.

Supplementary Table 2. Cohorts' distribution into microbial groups.

Group	Cohort I; *"Health"*	Cohort II; *"Disease stages"*
	n (%)	***n (%)***
CST I-A	69 (20)	38 (19)
CST I-B	29 (9)	19 (10)
CST II	8 (2)	9 (4)
CST III-A	52 (15)	28 (14)
CST III-B	18 (5)	21 (11)
CST IV-A	67 (20)	40 (20)
CST IV-B	65 (19)	32 (16)
CST IV-C	14 (4)	7 (3)
CST V	19 (6)	6 (3)
Total	341	200

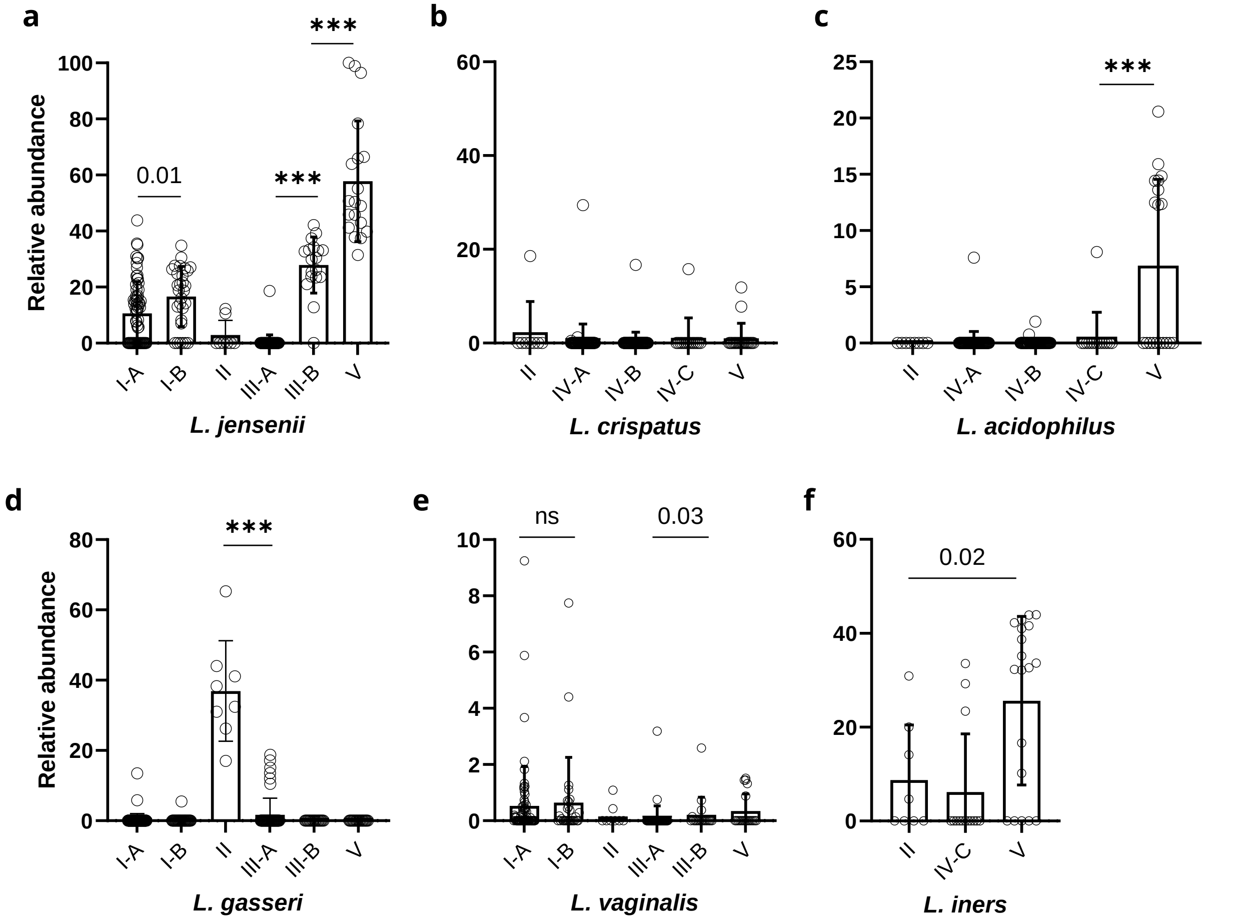

Supplementary Figure 1. Relative abundances for *Lactobacillus* species.

Analysis of relative abundances for *L. jensenii* (**a**), *L. crispatus* (**b**), *L. acidophilus* (**c**), *L. gasseri* (**d**), *L. vaginalis* (**e**) and *L. iners* (**f**) in CSTs (n = 341). *L. jensenii* exhibits a lower relative abundance in CSTs I-A, I-B, II, III-A, and III-B compared to V and a significantly higher relative abundance in CST III-B compared to III-A (**a**). There is a low relative abundance for *L. crispatus* in CSTs II, IV-A, IV-B, IV-C, and V (**b**). Likewise, the abundance for *L. acidophilus* is low in CSTs II, IV-A, IV-B, and IV-C, but higher in CSTs V (**c**). There is a significantly higher abundance for *L. gasseri* in CST II than in III-A (**d**). *L. vaginalis* exhibit very low abundance in all CSTs (**e**), whereas *L. iners* shows higher abundance in CST V (**f**). Error bars represent means ± s.d and differences in relative abundances were analyzed by using a Mann–Whitney U test followed by Bonferroni correction. *, p <0.006; **, p <0.001; ***, p <0.0001; ns = no significant. Source data are provided as a Source Data file.

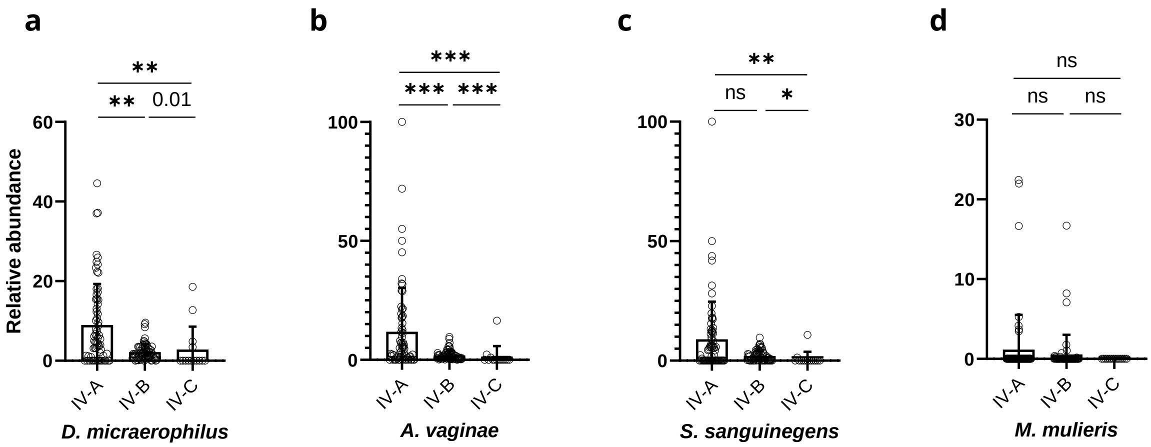

Supplementary Figure 2. **Relative abundances for bacteria in diverse-associated CSTs**.

Analysis of relative abundances for *D. micraerophilus* (**a**), *A. vaginae* (**b**), *S. sanguinegens* (**c**), and *M. mulieris* (**d**) in diverse-associated CSTs demonstrates the higher abundance for *D. micraerophilus* and *A. vaginae* in CST IV-A when compared to IV-B and in IV-B when compared to IV-C. Alternatively, the abundance for *S. sanguinegens* and *M. mulieris* remains unchanged in CSTs IV-A and IV-B, but for *S. sanguinegens* is higher in CST IV-B than in IV-C. Error bars represent means ± s.d and differences in relative abundances were analyzed by using a Mann–Whitney U test followed by Bonferroni correction. *, p <0.004; **, p <0.001; ***, p <0.0001; ns = no significant. Source data are provided as a Source Data file.

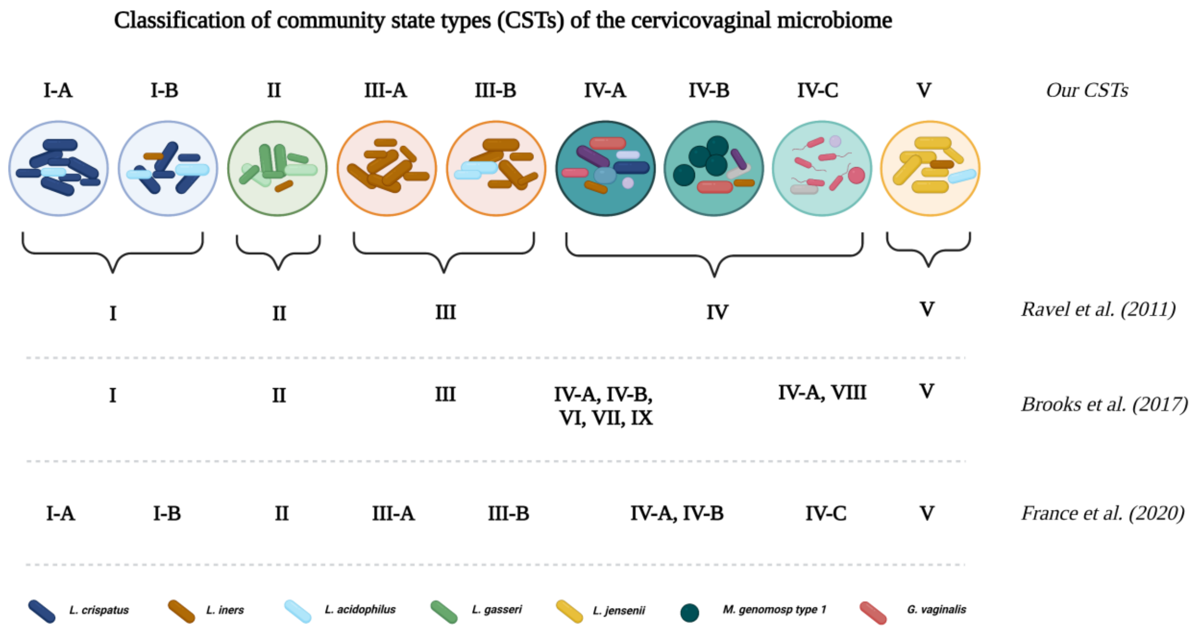

Supplementary Figure 3. Classification of CSTs of the CVM.

According to our classification, CSTs can be categorized into five major groups that resemble the initial classification reported by Ravel et al. (15). Additional and rare CSTs are assigned into these groups as subdivisions. Brooks et al. (19) described a very similar classification, with the major difference being the extension of CSTs up to nine major groups, which we combined into the well-known CSTs IV-A and IV-C. The classification by France et al. (21) is similar to ours, with the only difference being the microbial composition of CSTs IV-A and IV-B that is likely the result of their use of 16S rRNA gene sequencing for microbiome profiling. Created with BioRender.com.
